# Preferential Disomic Segregation and *C. micrantha/C. medica* Interspecific Recombination in Tetraploid ‘Giant Key’ Lime; Outlook for Triploid Lime Breeding

**DOI:** 10.3389/fpls.2020.00939

**Published:** 2020-06-25

**Authors:** Dalel Ahmed, Franck Curk, Jean Charles Evrard, Yann Froelicher, Patrick Ollitrault

**Affiliations:** ^1^ UMR AGAP, INRA, CIRAD, Montpellier SupAgro, Univ Montpellier, San Giuliano, France; ^2^ UMR AGAP, INRA, CIRAD, Montpellier SupAgro, Univ Montpellier, Montpellier, France; ^3^ CIRAD, UMR AGAP, San Giuliano, France

**Keywords:** polyploid, *C. aurantiifolia*, *C. latifolia*, meiosis, inheritance, crossing over, KASPar genotyping, single nucleotide polymorphism

## Abstract

The triploid ‘Tahiti’ lime (*C. x latifolia* (Yu. Tanaka) Tanaka) naturally originated from a merger between a haploid ovule of lemon (*C. x limon* (L.) Burm) and a diploid pollen from a ‘Mexican’ lime (*C. x aurantiifolia* (Christm.) Swing). The very limited natural inter-varietal diversity and gametic sterility of *C. latifolia* requires a phylogenomic based reconstruction breeding strategy to insure its diversification. We developed a strategy based on interploid hybridization between diploid lemon and the doubled diploid ‘Giant Key’ lime. This lime is a doubled diploid of ‘Mexican’ lime, itself a natural interspecific F1 hybrid between *C. medica* L. and *C. micrantha* Wester. For an optimized breeding program, we analyzed the meiotic behavior of the allotetraploid lime, the genetic structure of its diploid gametes, the interspecific recombination between *C. medica* and *C. micrantha*, and constructed its genetic map. A population of 272 triploid hybrids was generated using ‘Giant Key’ lime as pollinator. One hundred fifty-eight SNPs diagnostic of *C. micrantha,* regularly distributed throughout the citrus genome were successfully developed and applied. The genetic structure of the diploid gametes was examined based on *C. micrantha* doses along the genome. The diploid gametes transmitted in average 91.17% of the parental interspecific *C. medica*/*C. micrantha* heterozygosity. Three chromosomes (2, 8, and 9) showed disomic segregation with high preferential pairing values, while the remaining chromosomes showed an intermediate inheritance with a preferential disomic trend. A total of 131 SNPs were assigned to nine linkage groups to construct the genetic map. It spanned 272.8 cM with a low average recombination rate (0.99 cM Mb^-1^) and high synteny and colinearity with the reference clementine genome. Our results confirmed that an efficient reconstruction breeding strategy for ‘Tahiti’ lime is possible, based on interploid hybridization using a doubled diploid of *C. aurantiifolia*. The tetraploid parent should be selected for favorable agronomic traits and its genetic value should be efficiently inherited by the progeny thanks to transmission of the high level of parental heterozygosity. However, it would require developing numerous progeny to overcome the linkage drag caused by the limited interspecific recombination associated with the predominant disomic inheritance.

## Introduction

Polyploidy, the state of an organism having more than two paired set of chromosomes, is a major component of angiosperm evolution (Grant, 1981; Otto and Whitton, 2000; Wendel, 2000; Madlung, 2013; Soltis et al., 2015; Alix et al., 2017; Van de Peer et al., 2017) and polyploidization is considered to be the most common sympatric speciation mechanism (Otto and Whitton, 2000; Landis et al., 2018). Most plant evolutionists (Harlan and De Wet, 1975; Bretagnolle and Thompson, 1995; Ramsey and Schemske, 2002; Storme and Geelen, 2013) consider that unreduced (2n) gametes formation is the main mechanism of polyploidization. The benefits of polyploidy in long term evolution have been attributed to different factors, including mutation buffering, increased allelic diversity and heterozygosity, sub- or neo-functionalization of duplicated genes, epigenetic changes, and genome neo-regulation, resulting in phenotypic variation (Comai, 2005; Beest et al., 2012; Madlung, 2013). In several plant species, polyploidization has also been shown to immediately confer increased tolerance to different abiotic stresses including salt stress (Meng et al., 2011; Liu et al., 2011; Chao et al., 2013; Del Pozo and Ramirez-Parra, 2014; Del Pozo and Ramirez-Parra, 2015; Xue et al., 2015), drought (Liu et al., 2011; Manzaneda et al., 2012; Del Pozo and Ramirez-Parra, 2014; Del Pozo and Ramirez-Parra, 2015; Zhang et al., 2015), cold (Liu et al., 2011; Deng et al., 2012), and nutrient deficits (Deng et al., 2012). For several horticultural crops such as banana, grapes, watermelon, yams, and citrus, the triploid level appears to be optimum from an agronomical point of view or to produce seedless fruits. Some spontaneous triploid cultivars such as ‘Cavendish’ and ‘Plantain’ for banana or ‘Tahiti’ type for lime are essential ideotypes supporting the main worldwide production of these crops. Interploid hybridization involving diploid and tetraploid parents is a classical breeding approach to diversify these triploid crops.

Tetraploid meiosis behavior and particularly the transmission of parental heterozygosity greatly depends on the origin of the polyploid. Two extreme models are generally considered, disomic in allotetraploids and tetrasomic in autotetraploids (Stebbins, 1947; Stift et al., 2008; Sybenga, 2012). In allotetraploids resulting from the merger of the genomes of two divergent species, there are two sets of homologous chromosomes. Each chromosome pairs only with its homologous form during meiosis and only bivalents are formed (Stebbins, 1947). This results in disomic inheritance with 100% of the interspecific heterozygosity transmitted by each gamete (Stift et al., 2008). In autotetraploids, the presence of four homologous chromosomes instead of two results in equal opportunities to pair at meiosis, leading to tetrasomic inheritance with potential multivalent formation (Jackson and Jackson, 1996). In doubled diploids, this hypothetically leads to 66% restitution of heterozygosity (Sanford et al., 1983; Aleza et al., 2016), in the absence of double reduction (DR). In cases where the parents are divergent but have retained enough homology to prevent exclusive preferential pairing, intermediate inheritance patterns between di and tetrasomic are expected (Stebbins, 1947; Sybenga, 1996; Stift et al., 2008; Jeridi et al., 2012). Stift et al. (2008) developed a likelihood based approach to evaluate whether disomic, intermediate, or tetrasomic inheritances best fitted the segregation of genetic markers and to estimate preferential pairing and double reduction rates. The method was simplified for doubled diploids by Aleza et al. (2016).

The specificities of tetraploid meiosis require dedicated tools for genetic mapping. While several software tools are available to construct linkage maps of diploid species, including MapMaker (Lander et al., 1987), JoinMap (Stam, 1993; Jansen et al., 2001; Van Ooijen, 2006), R/qtl (Broman et al., 2003), OneMap (Margarido et al., 2007), MSTMAP (Wu et al., 2008), and ASMap (Taylor and Butler, 2017), the development of tools intended for the analysis of polyploids only began in recent years. It includes TetraploidMap (Hackett and Luo, 2003; Hackett et al., 2007), TetraploidSNPMap (TSNPM) (Hackett et al., 2017). R packages were recently developed to analyze polyploids such as {netgwas} (Behrouzi and Wit, 2017), {PERGOLA} (Grandke et al., 2017), and {MDSMap} (Preedy and Hackett, 2016; Preedy et al., 2018). The most recently released R package is {polymapR} (Bourke et al., 2016; Bourke et al., 2017b; Bourke et al., 2017a), which was created to construct genetic maps, and handles polysomic triploids resulting from a 4x × 2x cross, tetraploids, whether tetrasomic or with mixed meiotic pairing, segmental allotetraploids and hexaploids.

Most *Citrus* species and related genera are diploid with a basic chromosome number x = 9 (Krug, 1943). However some triploid and tetraploid plants were encountered in the citrus germplasm (Longley, 1925; Iwasaki, 1943; Jackson and Sherman, 1975). Cameron and Frost (1968) observed that 2.5% of nucellar seedlings from a broad range of citrus cultivars were tetraploid and proposed chromosome doubling of nucellar cells as the general mechanism of spontaneous tetraploidization. This was confirmed by SSR marker analysis in a wide range of spontaneous tetraploids (Aleza et al., 2011). Triploid citrus hybrids resulting from diploid crosses also appear to be relatively common. They mainly arise from 2n megagametophytes (Esen and Soost, 1971; Geraci et al., 1975). The restitution of the second division of the meiosis (SDR) appears to be the main mechanism behind the formation of 2n ovules (Cuenca et al., 2011; Xie et al., 2014; Cuenca et al., 2015; Aleza et al., 2015; Rouiss et al., 2017b). Ploidy manipulation and particularly triploid breeding became an important component of citrus genetics and breeding. Indeed, triploidy generally induces a high level of male and female sterility, which, when combined with parthenocarpy, leads to the production of seedless fruits (Ollitrault et al., 2008). Both sexual triploidization through 2n gametes (Ollitrault et al., 1994; Ollitrault et al., 2008; Aleza et al., 2010) and interploid crosses (Soost and Cameron, 1969; Starrantino and Recupero, 1981; Viloria and Grosser, 2005; Aleza et al., 2012a; Aleza et al., 2012b) have been widely exploited.

**Figure 1:**
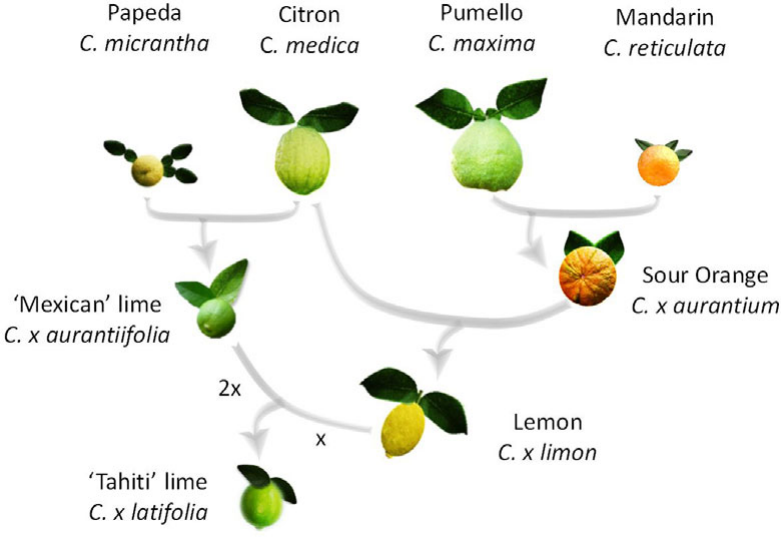
Origin of *C. latifolia*.

Lime is the only horticultural citrus group that includes diploid (‘Mexican’ lime type, *C. x aurantiifolia* (Christm.) Swing), triploid (‘Tahiti’ lime type *C. x latifolia* (Yu. Tanaka) Tanaka and ‘Tanepao’ lime type), and tetraploid (‘Giant Key’ lime) natural germplasm (Jackson and Sherman, 1975). It is also the one with the most complex phylogenomic structure. Indeed, recent molecular studies (Curk et al., 2016; Ahmed et al., 2019) demonstrated that the triploid ‘Tahiti’ lime type (including ‘Bears’ and ‘Persian’ lime cultivars) resulted from admixture of four ancestral taxa (*C. maxima* (Burm.) Merr., *C. medica* L., *C. micrantha* Wester and *C. reticulata* Blanco), while ‘Mexican,’ ‘Tanepao,’ and ‘Giant Key’ types involved only *C. medica* and *C. micrantha* gene pools. ‘Mexican lime’ types resulted from direct hybridization between *C. micrantha* and *C. medica* (Nicolosi et al., 2000; Curk et al., 2016; Wu et al., 2018) and ‘Giant Key’ lime is probably a doubled diploid of ‘Mexican’ lime type with two *C. micrantha* and *C. medica* allele doses all over its genome (Curk et al., 2016; Ahmed et al., 2019). *C. x latifolia* is assumed to have resulted from a single triploidization event by the fertilization of a lemon (*C. x limon* (L.) Burm) ovule by diploid pollen from *C. x aurantiifolia* (Curk et al., 2016; Ahmed et al., 2019; **Figure 1**). *C. x limon* resulted from a hybridization between *C. x aurantium* and *C. medica*, and *C. x aurantium* is itself an interspecific hybrid between *C. maxima* and *C. reticulata* (Curk et al., 2016; Wu et al., 2018).

Most of the lime export-market is based on *C. latifolia* ‘Tahiti’ lime type which has a very narrow genetic basis, and genetic diversification of the ‘Tahiti’ lime type is required to guarantee sustainable production. However, like in most species, the triploidy of ‘Tahiti’ lime is an evolutionary dead end due to male and female gamete sterility. Taking advantage of recent phylogenomic information, we developed a new breeding strategy with the aim of reconstructing the ‘Tahiti’ lime ideotype by interploid hybridization between diploid lemons and the tetraploid ‘Giant Key’ lime. Considering its interspecific *C. micrantha/C. medica* origin, the ‘Giant Key’ lime can be considered as an allotetraploid and a previous study using a few molecular markers of another doubled diploid ‘Mexican’ lime suggested a predominant disomic inheritance (Rouiss et al., 2018). However, the variability of the meiotic behavior along the genome and its consequences for interspecific recombination have not been characterized and no genetic maps of tetraploid limes have been published so far.

To optimize the reconstruction breeding strategy, the objectives of this work were (i) to analyze the meiotic behavior of the tetraploid ‘Giant Key’ lime and its impact on the genetic structure of the diploid gamete population, (ii) to implement its genetic map, and (iii) to analyze the interspecific recombination between *C. medica* and *C. micrantha* along the genome. To this end, SNPs diagnostic of *C. micrantha* (i.e. diallelic SNPs differentiating *C. micrantha* from all other *Citrus*) were selected from GBS data (Ahmed et al., 2019) and 131 markers based on allele specific competitive PCR were successfully developed and used to map the ‘Giant Key’ lime in a progeny of 272 triploid hybrids obtained by crossing diploid lemons and ‘Giant Key’ lime.

## Material and Methods

### Plant Material

Diploid lemons (‘Eureka’ ICVN 0100289, ‘Feminello’ ICVN0100180, ‘Frost lisbon’ ICVN 0100257, ‘Limoneira’ ICVN 0100197, ‘Santa Teresa’ ICVN0100179, ‘Corpaci’ ICVN0100191, ‘Villafranca’ ICVN0100193, ‘Berna’ ICVN0100345, and ‘AK’ ICVN 0100635) were fertilized with pollen of the tetraploid ‘Giant Key’ lime (ICVN 0100785). Among the seedlings, nucellar diploid plants (lemons present partial apomixes) and triploid hybrids were identified by flow cytometry. The meiotic behavior was analyzed and the genetic map of the tetraploid ‘Giant key’ lime was constructed using 272 selected triploid hybrids. In addition to the genitors, some varieties/species were also included in the study as controls at the genotype calling step: *C. micrantha* (ICVN 0101115), ‘Poncire commun’ citron (ICVN 0100701), respectively representative of homozygous genotypes of the *C. micrantha* and alternative alleles, ‘Mexican’ lime (ICVN 0100140) as heterozygous control with equivalent doses of *C. micrantha* and alternative alleles (1/1), ‘Tahiti’ lime (ICVN 0100058), and ‘Persian’ lime (ICVN 0101046) as representatives of the *C. latifolia* triploid lime ideotype, ‘Tanepao’ (ICVN0100836) and ‘Coppenrhad’ (ICVN010838) lime as representative of *C. aurantiifolia* triploid limes. All these varieties came from the collection of the Inra-Cirad Citrus Biological Resource Center in San-Giuliano, Corsica, France.

### Marker Development and Population Genotyping

Genetic analysis of polyploid populations requires an unambiguous estimation of allele doses for heterozygous genotypes. GBS analysis of polyploid citrus revealed the difficulty to infer allele doses at individual SNP polymorphisms (Ahmed et al., 2019). Therefore, the KASPar SNP genotyping method, allowing clear allele dose identification in triploid hybrids (Cuenca et al., 2013) was chosen to answer the research questions of this paper. At the phylogenomic level, the hybrids resulted from the following combination:

[(*C. maxima* x *C. reticulata*) x *C. medica*] x [C*. micrantha* x *C. medica*] with [*C. micrantha* x *C. medica*] parent being a doubled diploid (Ahmed et al., 2019). Therefore, specific alleles from *C. micrantha* (M) have the following segregation AA x MMAA where A is the alternative allele to the M one. They are therefore perfect markers to analyze the segregation of the tetraploid ‘Giant Key’ lime, construct its genetic map and to analyze the interspecific recombination between *C. micrantha* and *C. medica*. One hundred and eighty-nine diagnostic SNPs of *C. micrantha* (DSNPs) were selected from those identified from a GBS analysis which aimed at deciphering the mosaic genomes of citrus fruits (Ahmed et al., 2019). These DSNPs were also selected to be well distributed across the nine chromosomes and to be at more than 50 bases to the next identified polymorphism of the study of Ahmed et al. (2019).

The 272 triploid hybrids, their genitors and control varieties were genotyped for the 189 DSNP markers using KASPar by LGC Genomics (www.lgcgenomics.com). The KASPar™ genotyping system is a competitive, allele-specific dual Förster Resonance Energy Transfer (FRET)-based assay. LGC Genomics extracted the DNA from the leaf samples, designed the primers based on the SNP locus-flanking sequences, and performed the genotyping. Details on the KASPar method are provided in (Cuppen, 2007). The allele doses of the triploid hybrids and the tetraploid male parent were estimated from their respective allele signals based on the method described by Cuenca et al. (2013). The analysis was performed using KlusterCaller software (LGC Genomics) with manual identification of AAM and AMM heterozygous clusters. The data were then coded according to the number of *C. micrantha* doses, ranging from 0 to 2.

### Estimation of Preferential Pairing, Tau, and Double Reduction Parameters

Genotype calling was performed using KlusterCaller software (LGC Genomics). Under intermediate inheritance the marker segregation is directly dependent of the rates of preferential pairing and double reduction for the considered marker (Aleza et al., 2016). Using the data of the successful markers, we computed the following parameters:

(1) for each marker, parental heterozygosity restitution (PHR) was computed as the percentage of heterozygous diploid gametes. The estimation of PHR for each chromosome is the average of the values for all the markers on the chromosome.
(2) the tetrasomic parameter (τ) which defines the proportion of gametes resulting from random meiotic chromosomal pairing (Stift et al., 2008). A value of 1 indicates full tetrasomic inheritance characteristic of autotetraploids, while 0 corresponds to a fully disomic inheritance specific to allotetraploids. τ was computed using the maximum likelihood approach proposed for centromeric loci by Aleza et al. (2016) and adopted by Rouiss et al. (2018). Indeed in absence of recombination in the centromeric area and therefore a null value for double reduction, there is a direct relationship between τ and parental heterozygosity restitution for the centromeric markers (Aleza et al., 2016). For each chromosome, τ was calculated for four centromeric markers and the average value was considered. The chromosomal preferential pairing rate (PP), which defines the proportion of gametes resulting from exclusive pairing of homologous chromosomes and ranges between 0 and 1. PP was computed for each chromosome from the average values of τ as 1- τ.
(2) the double reduction parameter (β) indicating the frequency of double reductions relative to the total frequency of random meiotic associations (Stift et al., 2008). For fixed PP and τ values, the average β of three telomeric markers (when allowed by the centromere position) located at the beginning and at the end of each of the nine chromosomes was computed using the maximum likelihood approach (Aleza et al., 2016).

### Population Segregation and Diversity Analysis

Segregation distortion was evaluated through a χ^2^ test, first considering all the gametes and then only homozygous gametes. The markers displaying an excess of the *C. micrantha* allele relative to the Mendelian segregation were identified by calculating the difference between the observed and the theoretical (0.5) frequencies of the *C. micrantha* allele.

The proportion of the genome that derived from a *C. micrantha/C. medica* heterozygosity, *C. micrantha* or *C. medica* homozygosity were visually analyzed using GGT 2.0 software (van Berloo, 2008), which was also used to estimate the number of recombination events per individual.

Using the {ape} (Paradis and Schliep, 2018) R package, a neighbor-joining tree estimation (Saitou and Nei, 1987) was performed based on a dissimilarity matrix calculated as the Euclidean distances between each pair of markers.

### Mapping Analysis

The genetic map of the ‘Giant Key’ lime was mainly constructed using the {polymapR} R package (Bourke et al., 2017b), but also the {pergola} R package (Grandke et al., 2017) particularly during the linkage grouping stage. Genotyping data were filtered to less than 10% of missing data for both markers and hybrids. The matrix was also scanned to identify duplicate markers and individuals with redundant information. The genetic map of the ‘Giant key’ lime was constructed assuming tetrasomic segregation. The pairwise recombination frequencies were therefore computed using the random pairing option available in the {polymapR} R package. Both the {polymapR} and {pergola} packages were used for the linkage grouping stage. We made an initial linkage grouping using the independence LOD provided by {polymapR} score and confronted it to the hierarchical clustering analysis performed with {pergola}. The map was created using the multidimensional scaling algorithm and the Kosambi mapping function which allows incomplete interference among the recombination events. The graphical representation of the genetic map using the resulting ordered and spaced markers was generated by the MapChart program (Voorrips, 2002). Map distances were established in centiMorgan (cM). A Marey map plot was performed in Excel to evaluate colinearity between the genetic and physical positions of the markers (clementine reference genome; Wu et al., 2014).

## Results

### Marker Development

Based on GBS analysis of 53 varieties, 4 371 SNPs diagnostic of *C. micrantha* (DSNPs) were previously identified (Ahmed et al., 2019). From these, we selected 189 SNPs with no identified additional polymorphism at fewer than 50 bases, well distributed along the nine chromosomes and located on a gene whenever possible (**Supplementary Figure 1**). In this way, 22 SNPs were selected on chromosome 1 (C1), 24 on C2, 31 on C3, 19 on C4, 19 on C5, 19 on C6, 15 on C7, 17 on C8, and 23 on C9. The 189 sequences were sent to LGC Genomics to develop the primer set; information on the selected DSNPs is provided in **Supplementary Table 1**.

### Genotype Calling and Marker Analyses

After competitive, allele-specific PCR (KASPar methodology), genotype calling was performed for the 189 C*. micrantha* markers using KlusterCaller software (LGC Genomics) and the method proposed by Cuenca et al. (2013) for estimation of allele doses in polyploid plants.

**Figure 2:**
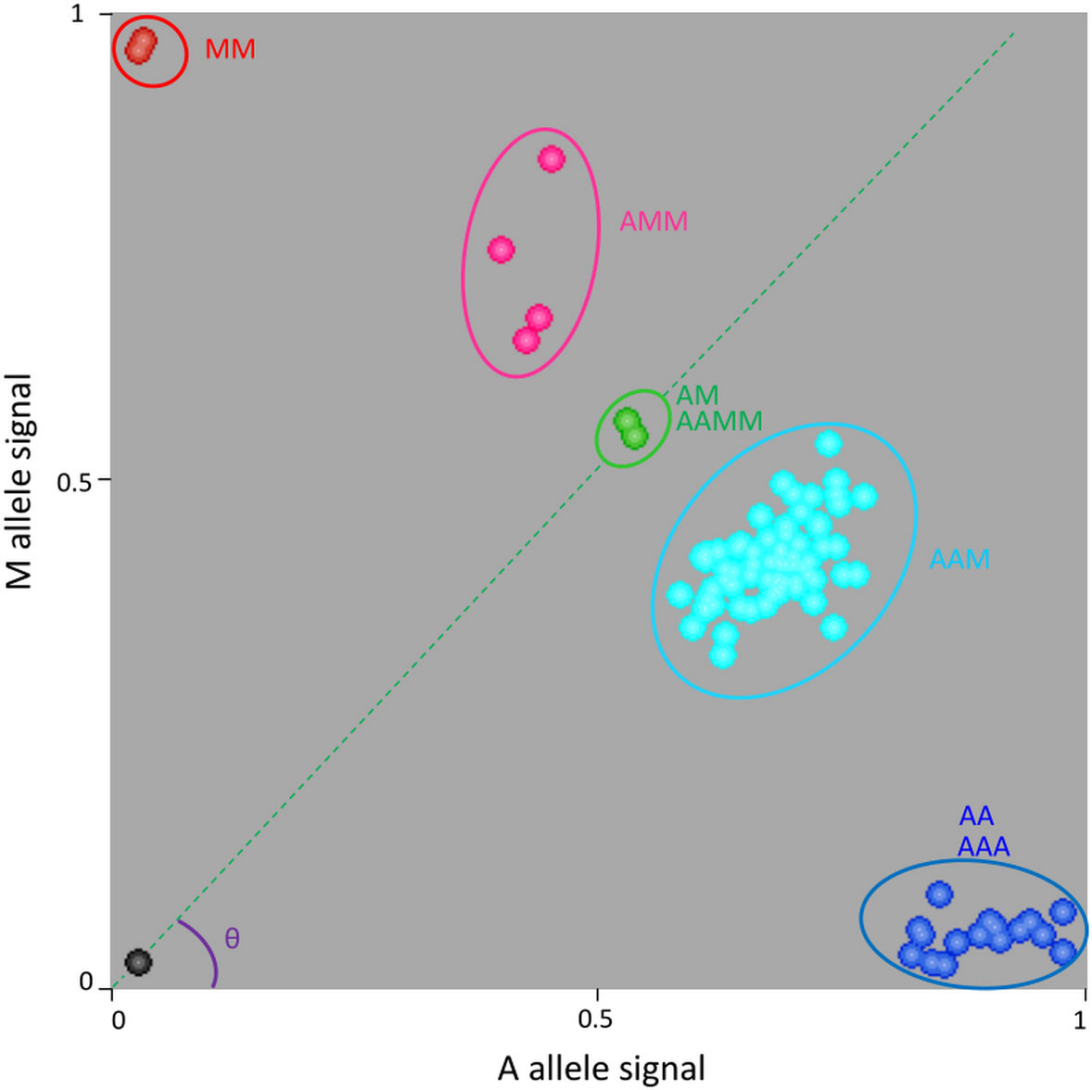
Example of genotype calling for the S03_46198875 SNP marker using KlusterCaller software and inference of allele dosage for triploid hybrids. M is the specific allele of *C. micrantha* and A is the alternative one. AA and MM, homozygous accessions; AM and AAMM, ‘Mexican’ lime and ‘Giant key’ lime respectively; AAM, triploid hybrids with one dose of *C. micrantha*; AMM, triploid hybrids with two doses of *C. micrantha*; AAA, triploid hybrids with no dose of *C. micrantha*.

One example is provided for marker S03_46198875 (**Figure 2**). Homozygous diploid controls were close to the x or y axes (*C. limon* and *C. micrantha* respectively homozygous for A and M alleles). ‘Mexican’ and ‘Giant Key’ limes had a heterozygous profile with a fluorescent signal for the two alleles and were used as the reference for equivalent A and M doses. Heterozygous triploid hybrids with a value of theta (θ) angle (angle between the x axis and the line joining the origin and the samples considered) higher than the one for ‘Mexican’/’Giant key’ lime samples were considered to have two M allele doses (AMM) while the one with a lower θ value was considered to have only one M dose (AAM). Therefore, cluster analysis enabled the identification of triploid hybrids (and corresponding diploid gametes) having zero, one, or two doses of M and allowed us to directly infer the genotype of the diploid gamete (zero, one, and two doses corresponding respectively to AA homozygous, AM heterozygous and MM homozygous).

Twenty-two SNPs failed genotyping and were removed from the analysis. The remaining 167 markers were filtered to less than 10% of missing data, which resulted in the removal of nine SNPs. As expected for *C. micrantha* diagnostic markers, the 158 selected markers displayed balanced heterozygosity for ‘Mexican’ and ‘Giant Key’ limes (AM and AAMM respectively) and homozygosity for lemons (AA). Among the 272 genotyped triploid hybrids, three were discarded due to a rate of missing data higher than 10%. Thus, a total of 158 SNPs and 269 hybrids were used in this work.

**Figure 3:**
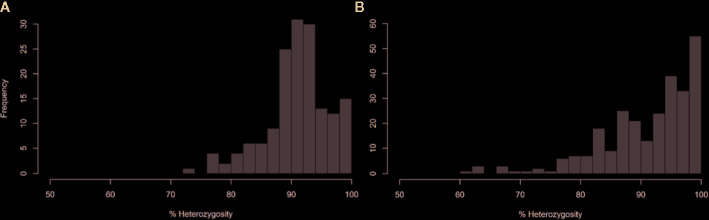
Distribution of heterozygosity rates among markers **(A)** and diploid gametes **(B)**.

The average of PHR over the whole genome was high (91.2%). The minimum values observed for markers and diploid gametes were 73.31 and 61.54%, respectively (**Figure 3**). Close to 64% of markers and 61% of gametes displayed more than 90% heterozygosity. Variations of PHR were observed between chromosomes (**Table 1**) ranging from 84.56% for chromosome 3 and up to 99.3% for chromosome 8. Among the diploid gametes, 8.55% displayed full interspecific heterozygosity restitution. All the chromosomes showed fully heterozygous 2n gamete percentages greater than 70%, except chromosomes 3 and 5, where only 50.19 and 55.39% of the diploid gametes respectively, were fully heterozygous. Very few gametes displayed full *C. micrantha* or *C. medica* homozygosity along a chromosome. Full *C. micrantha* homozygosity was observed only for chromosomes 5 and 7 (respective rates of 0.37 and 0.74%). Full *C. medica* homozygosity concerned chromosomes 1 (2.97%), 3 (1.49%), 6 (1.12%), and 7 (0.74%).

**Table 1:** Estimation of PHR of diploid gametes; percentage of gametes with full interspecific heterozygosity, *C. medica* or *C. micrantha* homozygosity.

Chromosome	PHR	Percentage of gametes with 100% restitution of		
		Interspecific heterozygosity	*C. micrantha* homozygosity	*C. medica* homozygosity
**Chr1**	90.26 ± 0.242	74.35	0	2.97
**Chr2**	92.58 ± 0.197	71.75	0	0
**Chr3**	84.56 ± 0.249	50.19	0	1.49
**Chr4**	92.04 ± 0.198	71	0	0
**Chr5**	85.78 ± 0.245	55.39	0.37	0
**Chr6**	89.61 ± 0.232	76.95	0	1.12
**Chr7**	90.34 ± 0.219	73.98	0.74	0.74
**Chr8**	99.3 ± 0.039	95.54	0	0
**Chr9**	96.21 ± 0.146	87.36	0	0
**Whole genome**	91.19 ± 4.622	8.55	0	0

PHR, parental heterozygosity restitution.

Analysis of the distribution of *C. micrantha* allele doses in the diploid gamete that generated the ‘Tahiti’ lime (**Supplementary Figure 2**) revealed that six chromosomes were fully heterozygous (1, 2, 4, 6, 8, 9), while homozygous markers were observed in the telomeric/sub-telomeric regions of chromosome 3, 5, and 7.

**Figure 4:**
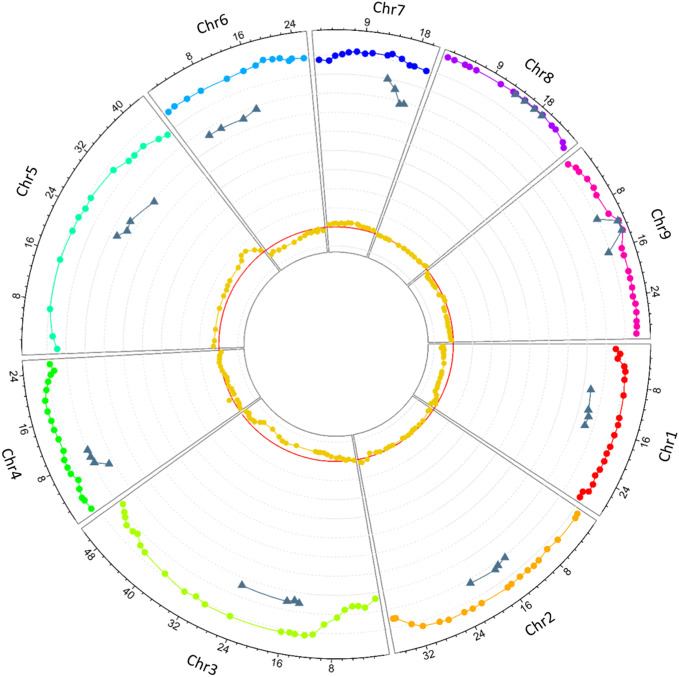
Circos plot of the heterozygosity rates of diploid gametes along the nine chromosomes (rainbow colors represent the nine chromosomes), the preferential pairing rates computed in centromeric loci (gray), and deviation from the expected frequency of *C. micrantha* allele (dark yellow). The X-axis represents the SNPs physical positions (Mb), the y-axis ranges from −0.1 to 1, each gray line stands for a unit; the red line stands for 0.

Allele segregation distortion was evaluated in the 158 markers. No significant distortion was observed in any of the markers, which can be explained by the high level of heterozygosity restitution that maintained each allele frequency close to 0.5. However, when only homozygous gametes were considered, significant distortions were observed. All the markers, except one located at the beginning of chromosome 3, displayed significant distortion (p < 0.05). One hundred and ten (69.62%) SNPs displayed an excess of the *C. medica* allele while 38 (24.05%) SNPs displayed an excess of the *C. micrantha* allele. All the markers on chromosomes 1, 6, and 9 on the one hand, and those on chromosomes 5 and 7 on the other hand were exclusively in excess of *C. medica* and *C. micrantha* alleles, respectively (**Figure 4**; **Table 2**). The segregation of 10 markers did not deviate significantly from the expected frequencies. They were distributed as follows: one SNP at the end of chromosome 4, seven successive markers located between 12.9 Mb and 21.9 Mb of chromosome 8, and two centromeric markers on chromosome 9 (**Figure 4**; **Table 2**). At the genotype level, except a SNP located at the beginning of chromosome 3 (S03_113190), all the others showed a deviation from the expected proportions that is hypothesized to result from a random chromosome pairing (1:4:1).

**Table 2:** Number of markers per chromosome with excess for *C. micrantha* or *C. medica* alleles; analysis based only on the homozygous gametes for the considered SNP.

Chromosome	Total number of markers	NDM	Markers in excess of	
			*C. micrantha* allele	*C. medica* allele
Chr1	19	0	0	19
Chr2	19	0	3	16
Chr3	25	0	1	24
Chr4	19	1	4	14
Chr5	14	0	14	0
Chr6	13	0	0	13
Chr7	14	0	14	0
Chr8	15	7	2	6
Chr9	20	2	0	18
Total	158	10	38	110

NDM, number of markers with no deviation from the expected allelic frequencies in homozygous gametes.

### Estimation of Preferential Pairing and τ Parameters

The disomic and tetrasomic inheritance behaviors of each chromosome were computed (**Figure 3**; **Table 3**) using the likelihood approach based on centromeric loci segregation (Aleza et al., 2016). All the chromosomes showed a predominant disomic inheritance. Preferential pairing (PP) varied among chromosomes. Chromosomes 8 and 9 had very high PP values, amounting to 0.995 and 0.945, respectively. The PP value of chromosome 2 was also high, 0.82. The remaining preferential pairing values ranged from 0.633 for chromosome 5 to 0.781 for chromosome 4, i.e. an intermediate segregation pattern with a preferential pairing trend. DR rates were estimated as proposed by Aleza et al. (2016) based on the rates of PP estimated from centromeric loci as being representative of the entire chromosome considered. A very low rate of DR was estimated for chromosome 6 (0.007), while estimated DR reached its maximum value (0.167) for chromosomes 3, 8, and 9 (**Table 3**).

**Table 3:** Estimation of PP and τ from centromeric loci and DR from telomeric/sub-telomeric loci of the nine chromosomes of ‘Giant Key’ lime.

Chromosome	PP	τ	DR
Chr1	0.751 ± 0.009	0.249 ± 0.009	0.136 ± 0.065
Chr2	0.82 ± 0.008	0.18 ± 0.008	0.118 ± 0.055
Chr3	0.749 ± 0.008	0.251 ± 0.008	0.167 ± 0
Chr4	0.781 ± 0.034	0.219 ± 0.034	0.084 ± 0.091
Chr5	0.633 ± 0.029	0.368 ± 0.029	0.109 ± 0.076
Chr6	0.669 ± 0.011	0.331 ± 0.011	0.007 ± 0.011
Chr7	0.733 ± 0.055	0.268 ± 0.055	0.132 ± 0.042
Chr8	0.995 ± 0	0.005 ± 0	0.167 ± 0
Chr9	0.945 ± 0.058	0.055 ± 0.058	0.167 ± 0

PP, preferential pairing; τ, tetrasomic rate; DR, double reduction.

### Genetic Mapping

**Figure 5:**
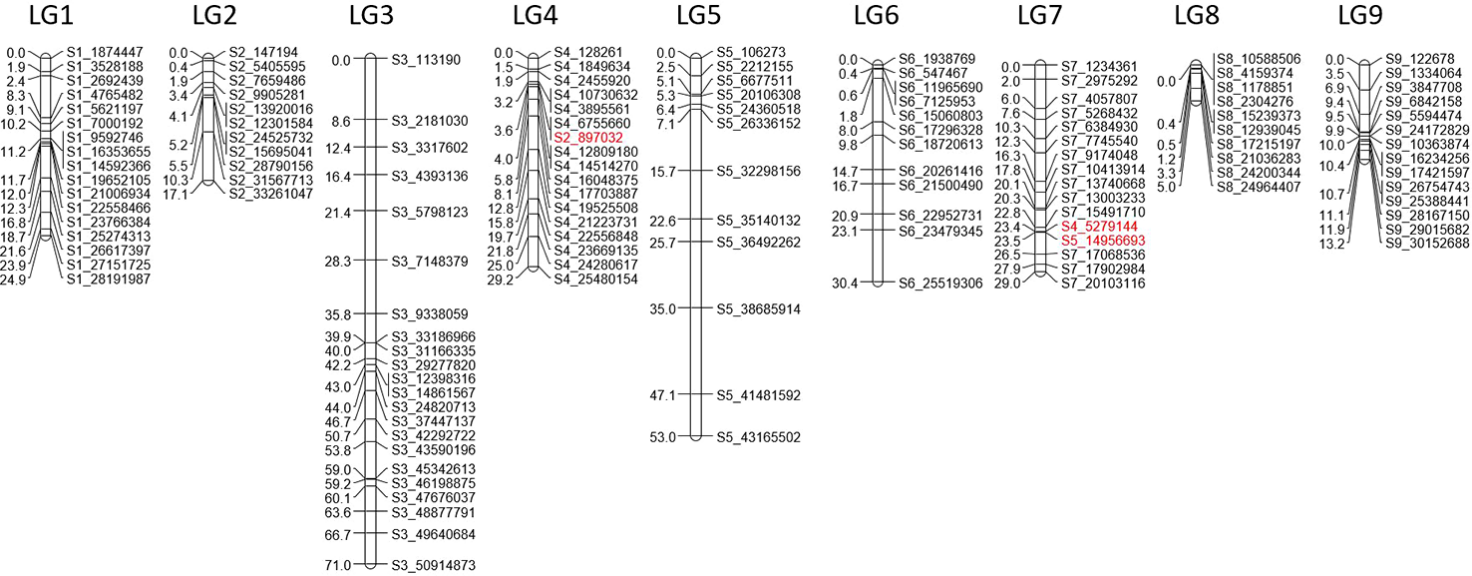
Genetic map of the ‘Giant Key’ lime. Red SNPs are the non syntenic ones.

From the filtered matrix composed of 158 SNPs, 269 hybrids and the two parents (lemon and ‘Giant key’ lime), 25 markers and 19 hybrids with redundant information were discarded using the R package {polymapR} (Bourke et al., 2017b) algorithm. Hence, the matrix used for genetic mapping was composed of 133 markers and 252 individuals, parents included. A LOD threshold of 14 in {polymapR} provided nine linkage groups as follows (**Supplementary Table 2**): LGs 1 to 7 grouped SNPs physically located on the corresponding chromosome number according to the clementine reference genome assembly. LG4 clustered an additional marker physically located on chromosome 2 of the clementine genome assembly, and LG7 included an SNP located on chromosome 4 and another on chromosome 5. LG8 included all the SNPs of chromosomes 8 and 9 while LG9 only grouped two SNPs of chromosome 2. We combined this result with the dendrogram (**Supplementary Figure 3**) obtained from the {PERGOLA} algorithm to set a suitable clustering. LG9 was deleted as the dendrogram revealed complete separation between the two SNPs composing it and the other clusters. LG8 was split into two LGs (named LG8 and LG9 on the dendrogram) according to the physical location of its SNPs, since the dendrogram displayed two separate clades each composed of SNPs on chromosomes 8 and 9. No changes were made to the other LGs. Both the {polymapR} clustering and the linkage grouping we used are summarized in **Supplementary Table 2**. A total of 131 markers out of 133 were assigned to a linkage group (**Figure 5**). Each of the nine LGs exhibited a set of markers physically located on the corresponding chromosome number except LG4 and LG7 as specified above. Considering the 131 mapped markers, overall synteny with the reference clementine genome is therefore high (97.7%). The total number of markers ranged between 10 for LG 8 and 22 for LG 3. The tetraploid ‘Giant Key’ lime map constructed under the tetrasomic inheritance hypothesis (all gametes are assumed to potentially undergo crossing over in the considered linkage group) spanned 272.8 cM (**Table 4**) with an average recombination rate of 0.99 cM Mb^-1^. It ranged from 0.21 to 1.54 cM Mb^-1^ for chromosomes 8 and 7 respectively (**Table 4**) closely linked with tetrasomic rates.

**Table 4:** Linkage group size (cM), genome size of the mapped chromosome parts (Mb), and average recombination rates per LG (cM/Mb-1) for tetraploid ‘Giant Key’ lime and tetraploid clementine.

Linkage group	Tetraploid ‘Giant Key’ lime			Tetraploid Clementine		
	LGS (cM)	GS (Mb)	RR	LGS (cM)	GS (Mb)	RR
LG1	24.9	26.32	0.95	95.5	27.96	3.42
LG2	17.1	33.11	0.52	108.5	28.33	3.83
LG3	71	50.8	1.4	143.3	44.39	3.23
LG4	29.2	25.35	1.15	63.3	21.86	2.90
LG5	53	43.06	1.23	91.5	35.54	2.57
LG6	30.4	24.97	1.22	68.1	16.98	4.01
LG7	29	18.87	1.54	28.3	6.87	4.12
LG8	5	23.79	0.21	77.7	22.64	3.43
LG9	13.2	30.03	0.44	76	29.72	2.56
Total	272.8	276.3	0.99	752.2	234.30	3.21

LGS, linkage group size; GS, genome size; RR, recombination rate.

**Figure 6:**
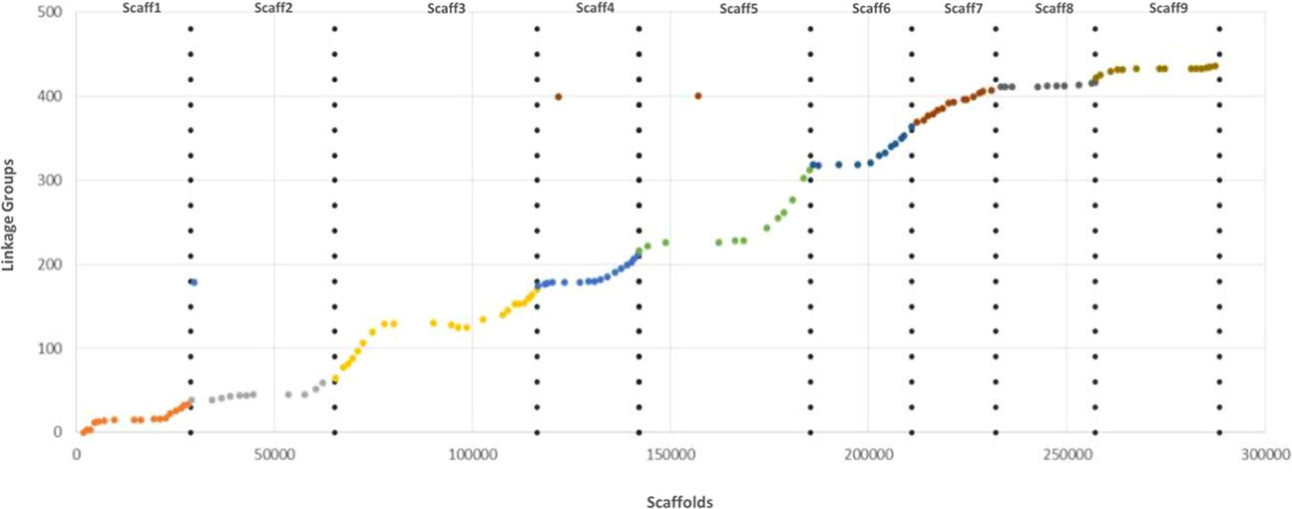
Marey map plot constructed with the 131 genetically mapped SNPs; x axis, physical positions on the clementine reference genome; y axis, positions on the ‘Giant Key’ lime genetic map.

Syntheny with the reference Clementine genome (Wu et al., 2014) was high with only three discording markers. The Marey map performed between physical positions of the 131 mapped markers over the clementine genome (Wu et al., 2014) and the genetic distances on each of the nine linkage groups (**Figure 6**) revealed high colinearity for all chromosomes with very slight inversions in LG3, LG4, LG6, and LG9.

To compare the recombination rate with a citrus with tetrasomic tendency, we built a genetic map of the tetraploid clementine using the same R packages and 59 markers published in Aleza et al. (2016). It spanned 752.2 cM. The recombination rate in the tetraploid clementine map was 3.21 cM Mb^-1^ in average and ranged from 2.56 to 4.12 cM Mb-1 for the analyzed genome segments of chromosome 9 and 7 respectively (**Table 4**).

### Genetic Structure of the Diploid Gametes of ‘Giant Key’ Lime

Graphical genotypes of the 250 diploid gametes used in genetic mapping were established using GGT 2.0 software (van Berloo, 2008). *C. micrantha/C. medica* heterozygosity was represented by an average of 86.5% of the entire genome, while *C. micrantha* and *C. medica* homozygosity accounted for respectively 6% and 6.5% of the gamete genomes. The estimated number of inter-subgenome recombinations in meioses producing diploid gametes ranged between 0 and 12 with an average value of 3.34. Eleven percent of the gametes did not display interspecific recombination, 44% had a maximum of two recombinations, and 97.6% displayed less than 9 recombination events (**Supplementary Figure 4**; **Supplementary Table 3**). At linkage group level, the proportion of gametes with no recombination varied from 51% for LG3 to 95% for LG8. The percentage of gametes displaying at least two crossing overs ranged between 0 and 25.2% for chromosomes 8 and 3 respectively (**Supplementary Table 4**).

**Figure 7:**
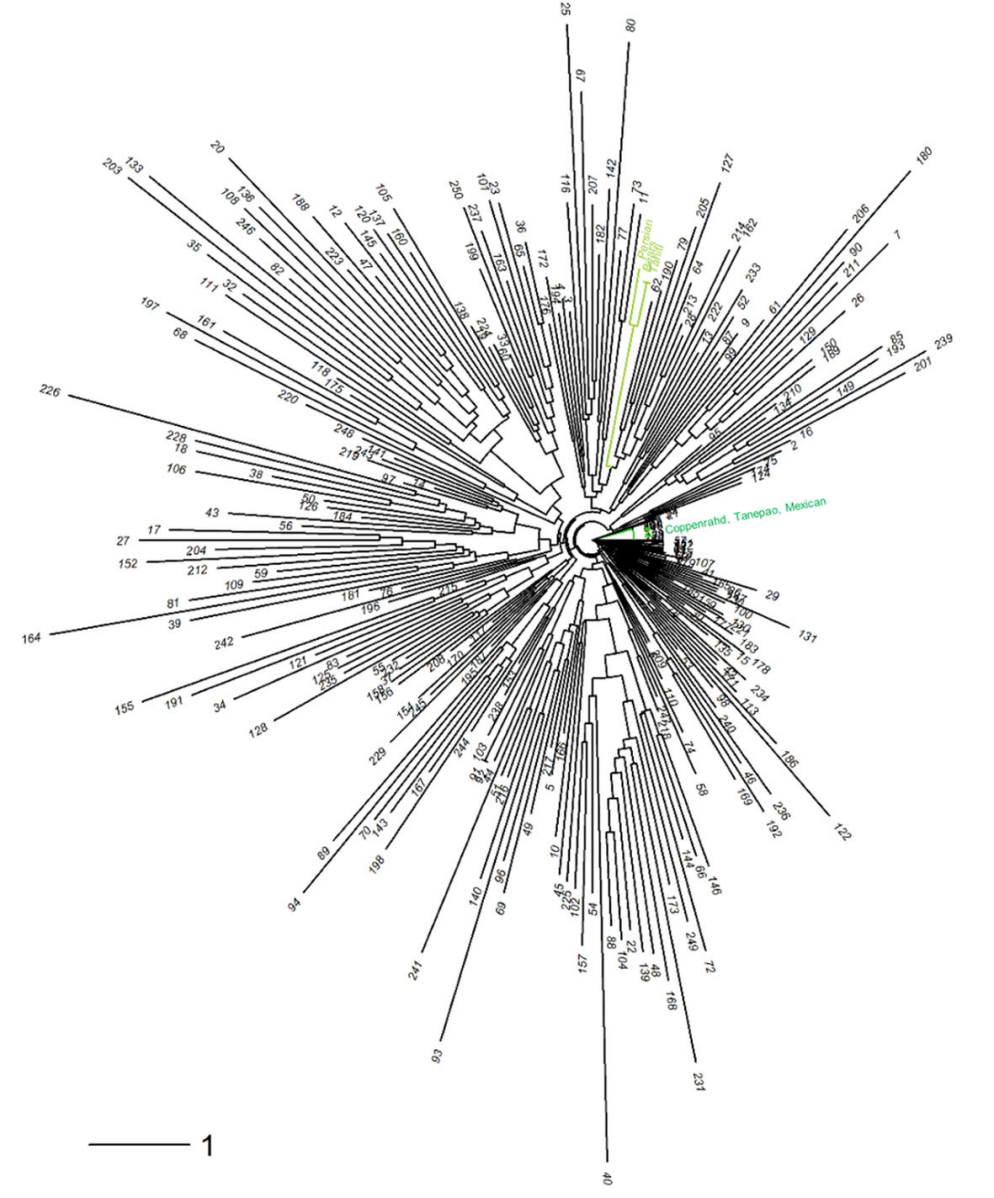
Neighbor-joining tree performed on the 250 triploid hybrids and six varieties of lime [‘Mexican’ lime, ‘Coppenrath’ lime, ‘Tanepao’ lime (dark green); ‘Tahiti’ lime, Persian lime, and ‘Bears’ lime (light green)].

The neighbor-joining analysis (**Figure 7**) of the diploid gametes revealed several groups, among which one cluster grouping ‘Tahiti,’ ‘Bears,’ and Persian limes, while ‘Coppenrahd,’ ‘Tanepao,’ and ‘Mexican’ limes were assembled together on another branch. Hybrid limes exhibited a large genotypic diversity among themselves, 25 were particularly close to the ‘Tahiti’ lime ideotype.

## Discussion

### *C. micrantha* DSNP Inheritance Reveals High Preferential Pairing Rates and Double Reduction Events (DR)

Inheritance patterns of molecular markers is a powerful method for determining meiotic behavior in polyploid species (Lerceteau-Köhler et al., 2003). It has been successfully used for several polyploid plant species including yellow cress (Stift et al., 2008), switchgrass (Okada et al., 2010), roses (Koning-Boucoiran et al., 2012), kiwi fruit (Wu et al., 2013), *mimulus* (Modliszewski and Willis, 2014), *chrysanthemum* (Klie et al., 2014), Bermuda grass (Guo et al., 2015), and citrus (Kamiri et al., 2011; Aleza et al., 2016; Kamiri et al., 2018; Rouiss et al., 2018).

Among the 189 SNPs diagnostic of *C. micrantha* selected from Ahmed et al. (2019)’s GBS data, 158 SNP competitive allele specific PCR markers were successfully developed. As expected, they were all heterozygous in ‘Mexican’ and ‘Giant Key’ limes resulting from interspecific hybridization (*C. micrantha* x *C. medica*) and homozygous in lemons. Efficient evaluation of allele doses with the method described by Cuenca et al. (2013) allowed us to infer the diploid gamete genotypes and hence to analyze the inheritance of the ‘Giant Key’ lime along the genome.

The analysis of the ‘Giant Key’ lime diploid gametes data revealed high preferential pairing values, highlighting a predominant disomic inheritance, especially for chromosomes 2, 8, and 9. The other chromosomes displayed a preferential pairing trend, and chromosome 5 had the lowest value (0.633). Such variations among chromosomes have already been described, for example in sugarcane (Jannoo et al., 2004) and autotetraploid pacific oysters (Curole and Hedgecock, 2005). They could reflect stochasticity, but also true differences in homology in different parts of the genome.

Preferential pairing values similar to those of ‘Giant Key’ lime were observed in the doubled-diploid ‘Mexican’ lime by Rouiss et al. (2018). Using a set of tetraploid hybrids between a colchicine-induced doubled-diploid ‘Clemenules’ clementine and a doubled-diploid ‘Mexican’ lime, these authors concluded a disomic segregation with high PP values for three linkage groups (LG2, LG7, and LG8), for intermediate segregation with a PP trend for five LGs (LG1, LG3, LG4, LG6, and LG9), and for intermediate segregation for LG5 (PP = 0.5). A similar tendency to disomic segregation was also observed for an intergeneric allotetraploid somatic hybrid between ‘Willow Leaf’ mandarin and *Poncirus trifoliata* cv ‘Pomeroy’ with PP values ranging from 0.53 to 0.91 (Kamiri et al., 2018). Conversely, the PP results reported by Kamiri et al. (2011) for *C. deliciosa* + *C. limon* allotetraploid somatic hybrids were lower, ranging between 0.05 and 0.71, as were those reported by Aleza et al. (2016) on the meiosis of the doubled-diploid clementine where significant PP was observed for only four out of nine LGs and did not exceed 0.55, thus largely exhibiting preferential tetrasomic segregation. Along with the preferential pairing rates, the parental heterozygosity restitution (PHR) of the ‘Giant Key’ tetraploid lime exhibited an average value of 91.2%, close to the average value estimated for the doubled-diploid ‘Mexican’ lime (Rouiss et al., 2018) and in the same range as the value observed for the nine chromosomes between ‘Willow Leaf’ mandarin and *Poncirus trifoliata* cv ‘Pomeroy’ ranging from 79 to 97%. These values are much higher than those reported for tetraploid somatic hybrids between *C. reticulata* and *C. limon* (64%) (Kamiri et al., 2011), between ‘Nova’ tangelo and ‘HB’ pummelo (76.2%) (Xie et al., 2015), or for doubled-diploid clementine (65%) (Aleza et al., 2016). Rouiss et al. (2018) proposed that the PP rate, and more generally meiotic behaviors, are determined by the phylogenomic structure of the genotypes involved. Citrus species are the result of reticulate evolution in which apomixes and vegetative propagation involve limited interspecific recombinations, therefore resulting in admixed mosaics of large genomic fragments (Nicolosi et al., 2000; Curk et al., 2014; Wu et al., 2014; Curk et al., 2015; Curk et al., 2016; Wu et al., 2018; Ahmed et al., 2019). The doubled-diploid ‘Mexican’ lime and ‘Giant Key’ lime have similar phylogenomic structures with two copies of *C. micrantha* and *C. medica* genomes, while three ancestors (*C. reticulata*, *C. maxima*, and *C. medica*) are part of the phylogenomic structure of the *C. reticulata* + *C. limon* somatic tetraploid hybrids (Kamiri et al., 2011), the ‘Nova’ tangelo + ‘HB’ pummelo is an admixture between *C. maxima* and *C. reticulata* (Xie et al., 2015), and the doubled-diploid clementine is mainly composed of a *C. reticulata* ancestor with a few fragments of *C. reticulata/C. maxima* heterozygosity (Aleza et al., 2016). It is therefore probable that there is greater differentiation between *C. micrantha* and *C. medica* chromosomes than between the other ancestors, particularly between *C. maxima* and *C. reticulata*, as also suggested by the genetic distances between these species estimated from GBS data (Ahmed et al., 2019).

In addition to preferential chromosome pairing, PHR transmission from the doubled-diploid parent is also affected by the double reduction (DR) rate, which varies according to the crossover distance between the locus and the spindle fiber (Mather, 1936). It is hypothesized to reach high values in telomeric chromosomal regions and to weaken and approach zero in the centromeric region (Welch, 1962; Butruille and Boiteux, 2000). In our study, DR rates were computed for telomeric markers with a fixed τ for the chromosome under consideration. The rates varied considerably between 0.007 and 0.167 (0.167 being the maximum possible frequency of the gamete when quadrivalents are formed and recombinant chromatids migrate to the same pole at anaphase I (Mather, 1936; Gallais, 2003; Stift et al., 2008). The DR rates we estimated for chromosomes 8 and 9 are questionable due to the very high level of preferential pairing and therefore the very low number of gametes resulting from tetrasomic inheritance. For chromosome 3, the notable drop in heterozygosity restitution (70%) observed in the telomeric part of the chromosome compared with the 90% in the centromeric area cannot only be explained by double reduction, suggesting a selective effect or possibly a variation in preferential pairing along the chromosome.

Bourke et al. (2017a) uncovered evidence showing that the strength of pairing can vary along a chromosome. This would add complexity to genetic analysis that generally assumes uniform pairing behavior along a chromosome. Previous reports on intra-chromosomal “mixosomy” concerned rainbow trout; the authors proposed possible “residual tetrasomy” (Allendorf and Danzmann, 1997) leading to variable pairing behavior along a chromosome, coupled with disomic segregation in the central regions. Similar behavior has also been reported in peanut (Leal-Bertioli et al., 2015; Nguepjop et al., 2016). Bourke et al. (2017a) suggested that “telomeric homology, where pairing initiation is thought to occur (Sybenga, 1975; Cifuentes et al., 2010), might have more influence than chromosome-length homology.”

Both sequence divergence and structural variations between *C. medica* and *C. micrantha* chromosomes probably drive the preferential pairing and intermediary preferential disomic inheritance observed for the tetraploid ‘Giant Key’ lime. The difference in PP rates between the chromosomes may be explained by variations of the extent of differentiation between the different sets of chromosomes as proposed by Stebbins (1950). Interestingly, none of the nine chromosomes of ‘Giant Key’ lime displayed tetrasomic inheritance, suggesting that pairing is more affected by overall differentiation rather than discrete and local large structural variations. Ongoing *de novo* sequencing projects of citrus species will provide decisive elements to understand the preferential pairing tendency and its variation between chromosomes.

### Variation in the Recombination Rate and Marker Ordering Between Genetic Maps of the Tetraploid ‘Giant Key’ Lime and Tetraploid Clementine

Despite predominant disomic inheritance, we observed interspecific recombinations in the nine citrus chromosomes. Their frequencies varied from 0.048 to 0.916 per gamete respectively for chromosomes 8 and 3, and were closely linked with random pairing values of the different chromosomes. The genetic mapping analysis revealed marked differences in the apparent recombination rates between the tetraploid ‘Giant Key’ lime maps (0.99 cM Mb^-1^) and those of the tetraploid clementine (3.21 cM Mb^-1^). The tetrasomic ‘Giant Key’ lime map spanned only 272.8 cM. The notable reduction in the recombination rate for the tetraploid lime compared to the tetraploid clementine is hypothesized to be a consequence of the ‘Giant Key’ inheritance behavior, which is close to disomic in three chromosomes (2, 8, and 9) and intermediate, with a disomic tendency, in the remaining ones, while the tetraploid clementine showed a mainly tetrasomic tendency (Aleza et al., 2016). The high preferential pairing strongly limits the proportion of gametes that can undergo interspecific chiasmata and consequently interspecific recombination.

In addition to the preferential disomic segregation behavior in tetraploid organisms, the divergence between parental genomes may play a key role in the variation in the recombination frequency of hybrids (Parker et al., 1982) and consequently in the size of LGs whatever the ploidy level. Several authors agree that sequence divergence at the interspecific level limits the sexual recombination at diploid level (Chambers et al., 1996; Liharska et al., 1996; Chetelat et al., 2000; Opperman et al., 2004; Li et al., 2006). In diploid citrus, such variation in recombination rates was found between clementine and sweet orange along six out of nine LGs and generated shorter LGs in sweet orange (Ollitrault et al., 2012). The authors suggested that these observations were associated with the interspecific heterozygosity of *C. reticulata/C. maxima*, which is higher in sweet orange than in clementine. Both the tetraploid ‘Giant Key’ lime and the ‘Mexican’ lime display a full heterozygous pattern between *C. medica* and *C. micrantha* species (Curk et al., 2016; Ahmed et al., 2019), while the clementine (*C. clementina*) rather displayed fragments of *C. reticulata/C. maxima* heterozygosity (Wu et al., 2014; Oueslati et al., 2017; Wu et al., 2018; Ahmed et al., 2019). It is possible that, in addition to the predominant disomic inheritance, the divergence between *C. medica* and *C. micrantha* genomic sequences contributes to the decrease in the observed recombination rates, even for gametes resulting from homologous pairing. Genetic mapping of the diploid ‘Mexican’ lime should allow this hypothesis to be tested.

In *Arabidopsis*, Pecinka et al. (2011) concluded that meiotic recombination between homologous chromosomes was more frequent in autotetraploids and allotetraploids than in diploid lines. A recent study (Pelé et al., 2017) concluded that *Brassica* allotriploid hybrids (AAC) showed 1.7 to 3.4 times more overall crossing overs between the homologous A chromosomes than diploid AA lines. Moreover, the authors observed a dramatic change in the crossing over pattern with homogeneous distribution along the genome in the triploid, while the diploid displayed a classical marked decrease in the extent of its pericentromeric areas. Chromosome interference was also strongly reduced in the allotriploids. In our case, the ‘Giant Key’ lime is a doubled-diploid of an F1 interspecific hybrid and therefore there is no intraspecific polymorphism between homologous chromosomes, and intraspecific recombinations are untraceable. Our conclusions on a strong reduction of the observed recombination compared with the reference clementine genetic map (Ollitrault et al., 2012) concern only interspecific recombination. Like on the reference genetic map of the diploid clementine (Ollitrault et al., 2012), we observed for the tetraploid ‘Giant Key’ lime a notable reduction in crossing overs in centromeric and pericentromeric areas.

Comparative analysis of the tetraploid ‘Giant Key’ lime with the physical positions of the markers used to perform mapping revealed globally good synteny and conserved marker ordering, except for some slight inversions in which only a few markers were involved (LG3, LG4, LG6, and LG9). The segregation distortion had very little effect on marker ordering (Hackett and Broadfoot, 2003). Therefore, the few differences observed could be due to marker translocations or inversions (Chen et al., 2008) but also to genotyping errors (Buetow, 1991; Hackett and Broadfoot, 2003), or even errors in the calculation of marker order (Curtolo et al., 2017) which could be due to a low accuracy of recombination estimates, especially in chromosomes with a high PP. Therefore, the genetic mapping of ‘Giant Key’ lime provides no evidence of the large heterozygous inversion described in cytogenetic studies of the diploid ‘Mexican’ lime (Iwamasa, 1966).

### Implication for the Reconstruction Breeding Strategy of the Triploid Lime Ideotype

‘Tahiti’ lime classified as *C. latifolia* is a highly productive variety with researched organoleptic and pomologic qualities, such as specific aromatic profile, acidity/sugar ratio, seedlessness, fruit size, and moderate peel thickness. It is the *Citrus* variety most tolerant to the Huanglongbing disease (the most devastating citrus disease caused by the bacteria *Candidatus Liberibacter* sp.). However, it displays high content of furanocoumarin potentially harmful for human health, its production period is limited and the genetic basis of this horticultural group is very low. The main medium term objective of this study is to design a reconstruction-breeding scheme for the sterile triploid ‘Tahiti’ lime ideotype. Several studies have shown that an important part of the actual phenotypic diversity of edible citrus should be related to the differentiation between the ancestral taxa before reticulation and introgression processes and that the structures of the phenotypic and genetic diversities are closely correlated. Such correlations with genetic structure were observed for morphological and pomological characters (Barrett and Rhodes, 1976; Ollitrault et al., 2003), flavone constitution (Mizuno et al., 1991), peel oil volatile compounds (Liu et al., 2013b), carotenoid contents (Fanciullino et al., 2006), coumarin and furanocoumarin constitution (Dugrand-Judek et al., 2015), and fingerprinting of secondary metabolites (Matsukawa and Nito, 2017). We therefore hypothesize that the selection of new hybrids with close phylogenomic structures, all along the genome, to the ones of the actual ideotypes of horticultural groups should also display close phenotypes for many traits.

The reconstruction breeding strategy for ‘Tahiti’ ideotype is based on a recent study by Curk et al. (2016) suggesting that ‘Tahiti’ lime is an interspecific hybrid resulting from the fusion of a haploid lemon (*C. limon*) ovule and a diploid pollen of a ‘Mexican’-like lime (*C. aurantiifolia*). Research on citrus phylogenomic structures by Ahmed et al. (2019) confirmed this hypothesis. The diploid pollen of the ‘Mexican’-like lime is assumed to result from an unreduced gamete of a diploid parent or from the meiosis of a tetraploid parent (Curk et al., 2016; Rouiss et al., 2018; Ahmed et al., 2019). The tetraploid ‘Giant Key’ lime in which we were interested was newly assumed to be a natural duplication of a ‘Mexican’ lime type chromosome, itself derived from a natural *C. micrantha* x *C. medica* hybridization event (Curk et al., 2016; Ahmed et al., 2019).

Under Curk et al. (2016)’s hypothesis concerning the origin of ‘Tahiti’ lime, the average restitutions of the *C. medica/C. micrantha* heterozygosity of Mexican lime, estimated from DSNP marker analysis (Curk et al., 2016) and GBS data (Ahmed et al., 2019) were respectively, 95.1 and 84.7%. These high PHR values invalidate the hypothesis of an unreduced gamete resulting from an SDR mechanism associated with an average PHR of 30–40% (Barone et al., 1995; Douches and Maas, 1998; Dewitte et al., 2012; Aleza et al., 2016). If the diploid gametes have an unreduced origin, the FDR mechanism seems to be a more plausible hypothesis since it transmits 70–80% of the parental heterozygosity (Barone et al., 1995; Douches and Maas, 1998; Dewitte et al., 2012; Aleza et al., 2016) and was described as being at the origin of diploid pollen in citrus hybrids (clementine x sweet orange) (Rouiss et al., 2017a). The pattern of *C. micrantha* doses along ‘Tahiti’ lime genome revealed that the diploid gamete that generated ‘Tahiti’ had homozygous segments only on telomeric areas of three chromosomes. This remains compatible with an FDR origin in which the 2n gametes are heterozygous from the centromere until the first crossing over.

Concerning the ‘Giant Key’ lime, the present study revealed an average PHR of 91.2%, close to that detected for the doubled-diploid ‘Mexican’ lime (Rouiss et al., 2018). Interestingly, analysis of the pattern of *C. micrantha* doses along the ‘Tahiti’ lime genome showed that the diploid gametes at the origin of ‘Tahiti’ lime were fully heterozygous for six chromosomes, while few homozygous loci (and therefore the evidence for interspecific recombination) were observed for three chromosomes (3, 5, 7) with relatively high rates of tetrasomic inheritance (respective τ values of 0.25, 0.37, and 0.27). All these results are fully compatible with the ‘Tahiti’ lime originating from an interploid cross, as already highlighted by Rouiss et al. (2018). Even if the FDR hypothesis remains valid, the meiotic behavior of the ‘Giant Key’ lime fits perfectly for a reconstruction breeding strategy of the ‘Tahiti’ lime ideotype. Moreover, in Corsican conditions, the ‘Giant Key’ lime has much higher pollen viability (> 60%) than the diploid ‘Mexican’ lime (< 15%) and interploid breeding strategy is more efficient than selecting rare spontaneous 2n gamete events to generate large triploid progenies (Aleza et al., 2012a).

Like the tetraploid ‘Giant Key’ lime, other doubled diploids of varieties of *C. micrantha/C. medica* interspecific origin such as ‘Mexican’ lime, *C. macrophylla*, *C. excelsa*, or *C. aurata* (Curk et al., 2016; Ahmed et al., 2019) should play a key role in implementing a reconstruction breeding strategy for the ‘Tahiti’ lime ideotype. Whether the interploid hybridization strategy is 4x × 2x or 2x × 4x, it should produce a progeny that inherits a large proportion of the genetic value of the tetraploid parent, due to the high level of PHR, but also allow more efficient breeding programs than sexual polyploidization, as searching for triploid hybrids resulting from rare unreduced gametes will no longer be required. Therefore, the doubled-diploid parent should be selected for favorable agronomic traits such as quality, resistance/tolerance to disease or adaptation to biotic and abiotic stresses. Considering that the preferential chromosome pairing should be very similar for pollen and ovules generation, and therefore the diploid gamete structures very similar, the choice between 4x × 2x or 2x × 4x strategies, with a same tetraploid parent, will be principally defined by parameters such as female and male fertilities and the rate of zygotic seedlings in progenies of the two parents. In case of *C. aurantiifolia/C. limon* combinations the relative higher rate of zygotic seedlings for *C. limon* make the *C. limon x C. aurantiifolia* option better.

The diploid lemon parent should be natural mutants of the common Mediterranean lemon or genotypes derived from *C. aurantium* x *C. medica* hybridization (Curk et al., 2016). Phylogenomic structures revealed by GBS analysis as well as the delivery of set of diagnostic markers of the four ancestral taxa (Ahmed et al., 2019) will help select a suitable lemon-like parent.

While the preferential disomic inheritance favors the transfer of a significant part of the genetic gain obtained during the tetraploid parent selection to its progeny, the limited interspecific recombination expands the genetic drag and will hamper the breeding process. Overcoming this issue will require the production of large hybrid populations, as emphasized by Rouiss et al. (2018). Leal-Bertioli et al. (2018) successfully increased diversity in peanuts through polyploid hybridization and homoeologous recombination and highlighted the potential of segmental allotetraploids for plant breeding.

## Conclusion

One hundred and fifty-eight SNP competitive allele-specific PCR markers, diagnostics of *C. micrantha* and regularly distributed over the citrus genome, were successfully developed. For the diploid lemon x tetraploid lime progeny considered here, they allowed the direct inference of diploid gametes of the ‘Giant Key’ lime by genotyping triploid hybrids. The tetraploid ‘Giant Key’ lime displayed mainly disomic segregation, although variation was observed among chromosomes. Chromosomes 2, 8, and 9 revealed the greatest preferential pairing values while the other chromosomes displayed intermediate segregation with a disomic tendency. Potential variation in PP along chromosome 3 is also suggested. Because of preferential disomic inheritance, a high PHR (*C. micrantha/C. medica*) was found along the nine chromosomes and the observed interspecific recombinations between the constitutive *C. medica* and *C. micrantha* genomes were strongly limited in comparison with the reference clementine genetic map. It was nevertheless sufficient to construct a genetic map of a tetraploid lime that revealed for the first time high synteny and colinearity with the clementine reference genome. The phylogenetic structure of the diploid gametes generated by the tetraploid lime parent reinforces the hypothesized interploid hybridization origin of the ‘Tahiti’ lime. Such a mechanism simplifies the implementation of extensive breeding reconstructing programs in which the high parental heterozygosity restored to the diploid gametes would lead to the creation of hybrids phenotypically close to the ‘Tahiti’ ideotype.

## Data Availability Statement

All datasets generated for this study are included in the article/**Supplementary Material**.

## Author Contributions

PO and YF designed the experiment. YF and JE developed the triploid progeny. DA and FC selected and developed the diagnostic SNP markers. DA and PO analyzed the data and wrote the manuscript.

## Funding

This work received financial support from the European Regional Development Fund under the framework PO FEDER-FSE Corse 2014-2020 number 247SAEUFEDER1A, project called Innov’Agrumes (ARR-18/517 CE, synergie number: CO 0009083). We thank also the Collectivité de Corse for the grant of DA (number ARR-15.036680.SR).

## Conflict of Interest

The authors declare that the research was conducted in the absence of any commercial or financial relationships that could be construed as a potential conflict of interest.
