# Supplementary material for: Preferential Disomic Segregation and C. micrantha/C. medica Interspecific Recombination in Tetraploid ‘Giant Key’ Lime; Outlook for Triploid Lime Breeding
Source: Front Plant Sci. 2020 Jun 25;11:939. doi: 10.3389/fpls.2020.00939 (PMC7330052; doi:10.3389/fpls.2020.00939)
Supplement: Supplementary file 1 [file DataSheet_1.pdf]

Ahmed D., Curk F., Froelicher Y. and Ollitrault P. 2020.Preferential disomic segregation and *C. micrantha / C. medica* interspecific recombination in tetraploid 'Giant Key' lime; outlook for triploid lime breeding. Frontiers in Plant Science.

Supplementary Figures 1 to 4

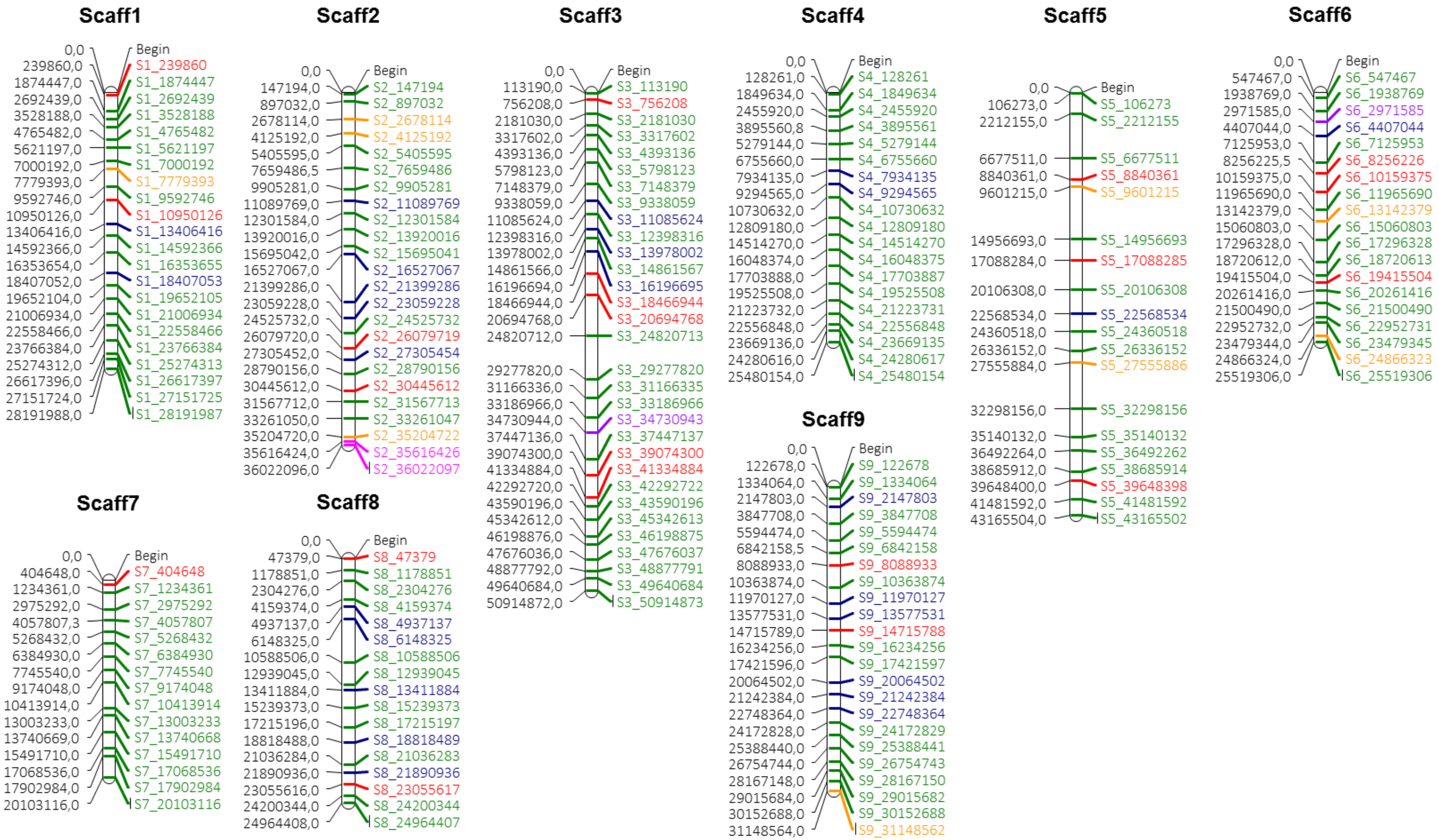

Supplementary Figure 1: Distribution of the 189 SNPs along the nine chromosomes. Red SNPs failed genotyping (20); orange SNPs had %NA >10% (9); purple SNPs displayed too high heterozygosity (2); blue SNPs had redundant information (25); magenta SNPs were discarded from mapping (2); green SNPs were assigned to a linkage group in the 'Giant Key' lime map (131).

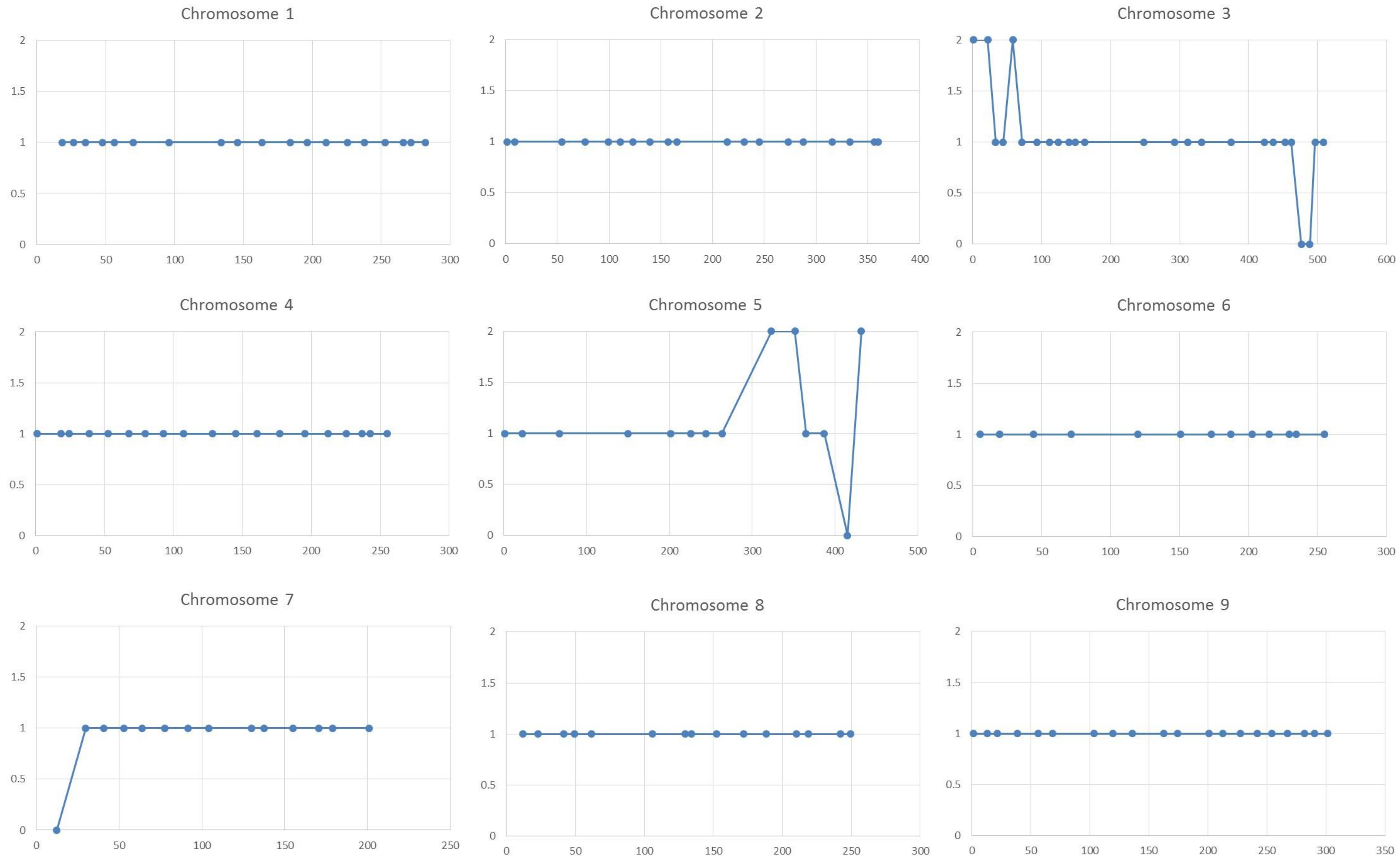

Supplementary Figure 2: Distribution of *C. micrantha* allele doses along the nine chromosomes of the diploid gamete of *C. aurantiifolia* that produced 'Tahiti' lime (1 correspond to interspecific parental heterozygosity restitution)

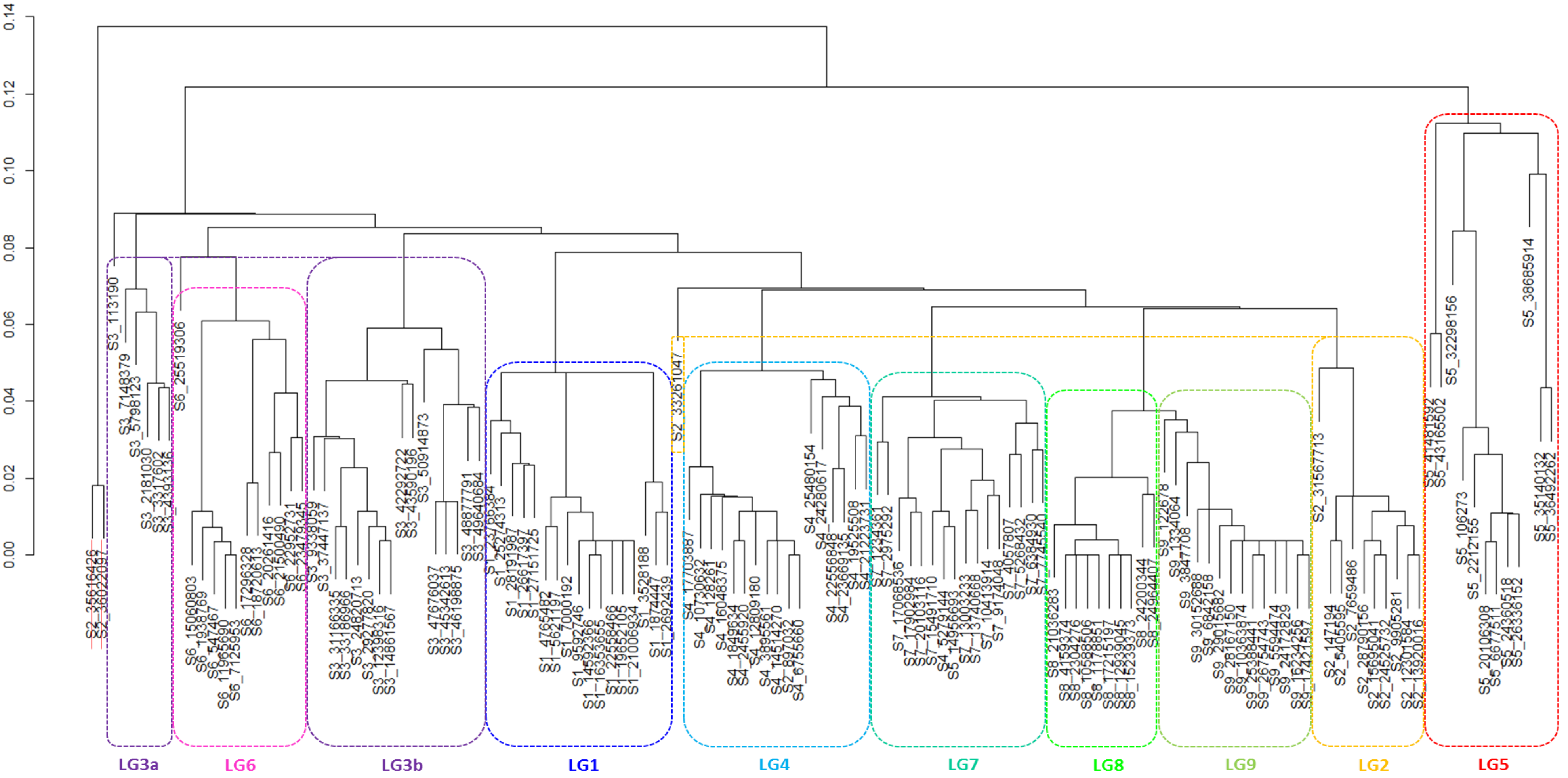

Supplementary Figure 3: Dendrogram performed at the linkage grouping (LG) step with the R package {PERGOLA} and the nine LGs used for genetic mapping.

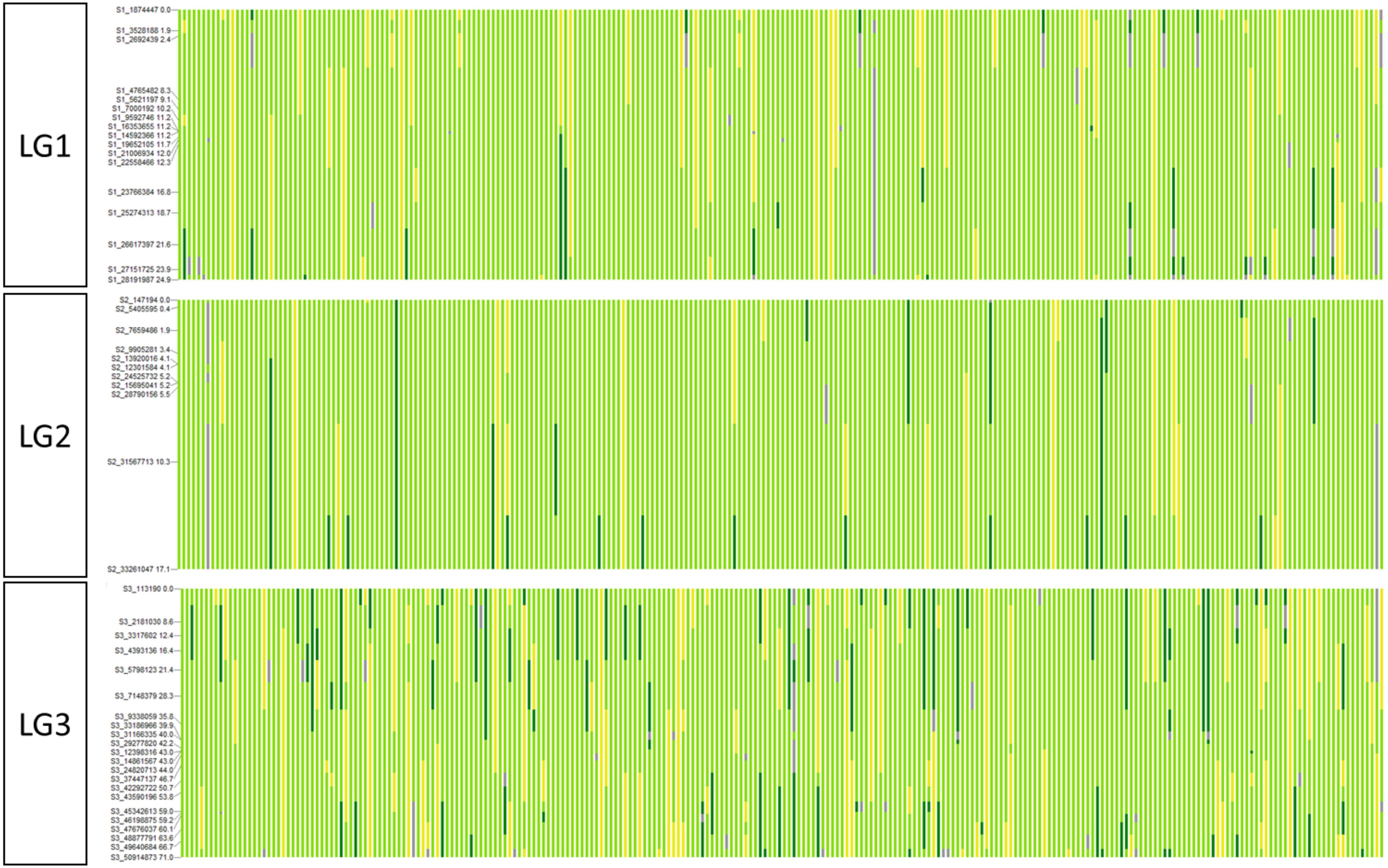

Supplementary Figure 4: Genomic structure of the diploid gametes along the nine linkage groups. Dark green: *C. micrantha* homozygosity; yellow: *C. medica* homozygosity; light green: *C. micrantha* / *C. medica* heterozygosity; grey: indeterminacy.

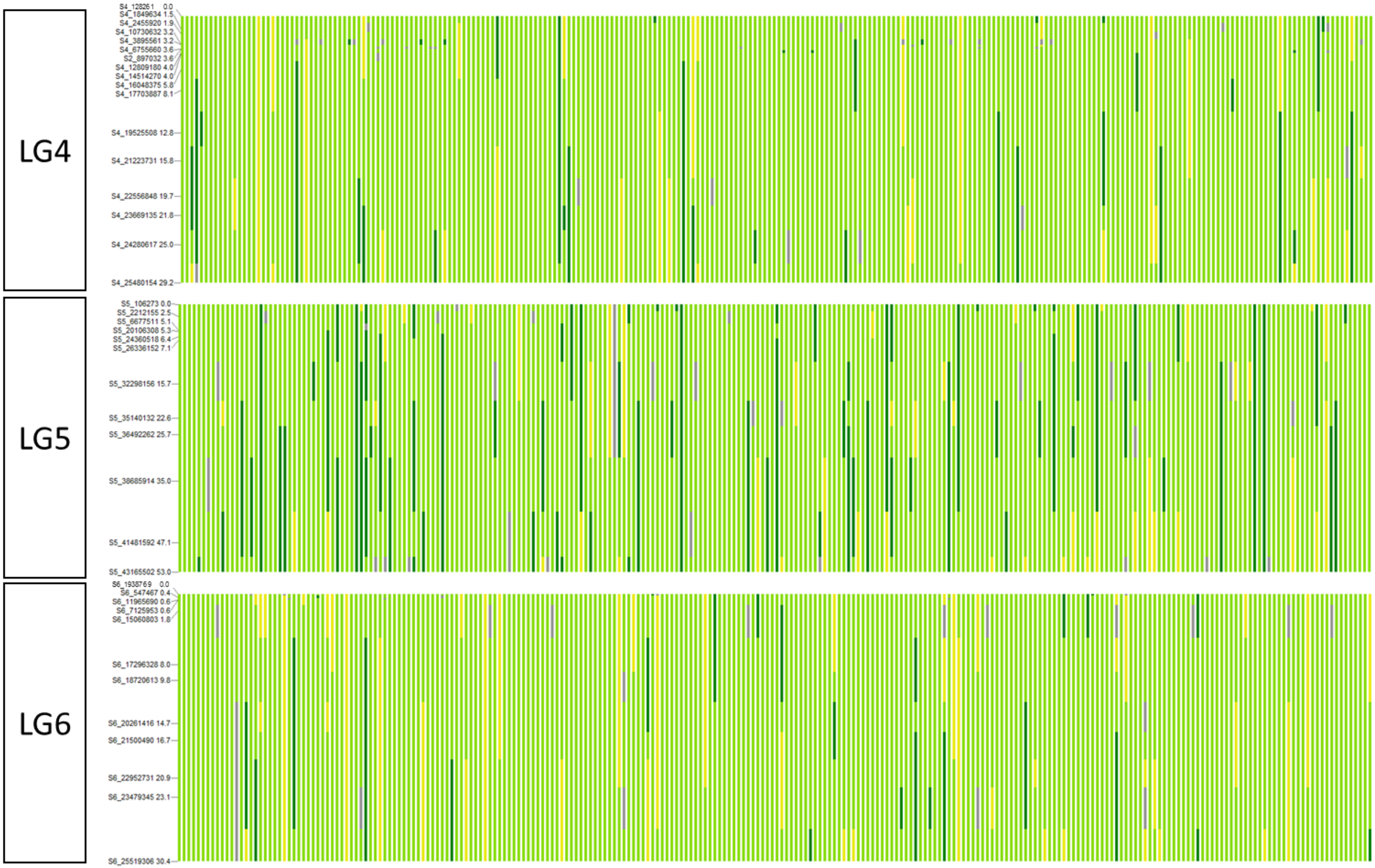

Supplementary Figure 4 (suite): Genomic structure of the diploid gametes along the nine linkage groups. Dark green: *C. micrantha*6 homozygosity; yellow: *C. medica* homozygosity; light green: *C. micrantha* / *C. medica* heterozygosity; grey: indeterminacy.

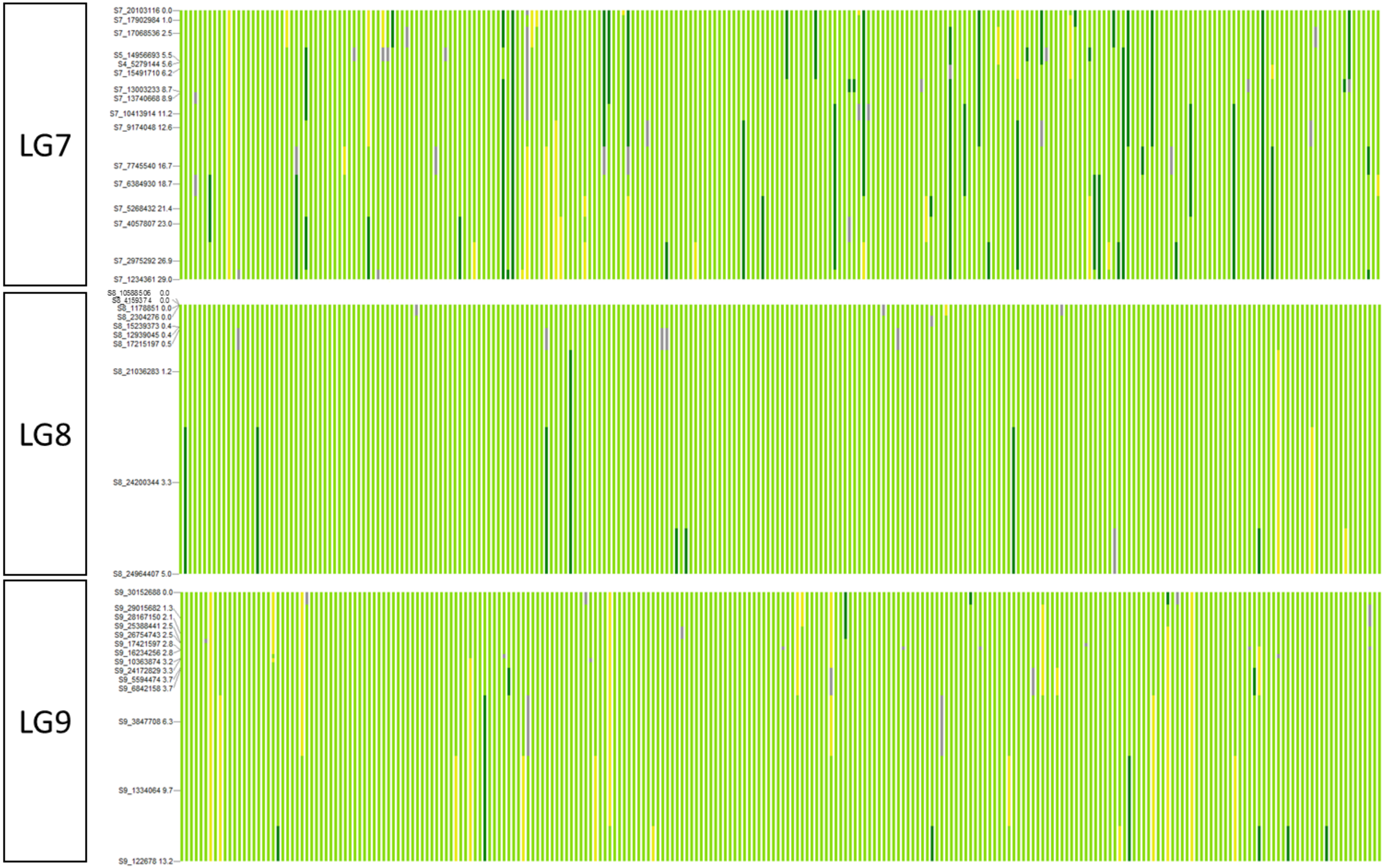

Supplementary Figure 4 (suite): Genomic structure of the diploid gametes along the nine linkage groups. Dark green: *C. micrantha*7 homozygosity; yellow: *C. medica* homozygosity; light green: *C. micrantha* / *C. medica* heterozygosity; grey: indeterminacy.
